# Treatment resistance analysis reveals GLUT‐1‐mediated glucose uptake as a major target of synthetic rocaglates in cancer cells

**DOI:** 10.1002/cam4.4212

**Published:** 2021-09-20

**Authors:** Julia Sieber‐Frank, Hans‐Jürgen Stark, Simon Kalteis, Elena‐Sophie Prigge, Richard Köhler, Carolin Andresen, Thomas Henkel, Georg Casari, Tobias Schubert, Wolfgang Fischl, Min Li‐Weber, Peter H. Krammer, Magnus von Knebel Doeberitz, Jürgen Kopitz, Matthias Kloor, Aysel Ahadova

**Affiliations:** ^1^ Department of Applied Tumor Biology Institute of Pathology University of Heidelberg Heidelberg Germany; ^2^ Collaboration Unit Applied Tumor Biology German Cancer Research Center (DKFZ) Heidelberg Germany; ^3^ IMAX Discovery GmbH Konstanz Germany; ^4^ Haplogen GmbH Vienna Austria; ^5^ Tumor Immunology Program German Cancer Research Center (DKFZ) Heidelberg Germany

**Keywords:** cancer biology, colorectal cancer, drug design, metabolic studies, molecular biology

## Abstract

Rocaglates are natural compounds that have been extensively studied for their ability to inhibit translation initiation. Rocaglates represent promising drug candidates for tumor treatment due to their growth‐inhibitory effects on neoplastic cells. In contrast to natural rocaglates, synthetic analogues of rocaglates have been less comprehensively characterized, but were also shown to have similar effects on the process of protein translation. Here, we demonstrate an enhanced growth‐inhibitory effect of synthetic rocaglates when combined with glucose anti‐metabolite 2‐deoxy‐D‐glucose (2DG) in different cancer cell lines. Moreover, we unravel a new aspect in the mechanism of action of synthetic rocaglates involving reduction of glucose uptake mediated by downregulation or abrogation of glucose transporter GLUT‐1 expression. Importantly, cells with genetically induced resistance to synthetic rocaglates showed substantially less pronounced treatment effect on glucose metabolism and did not demonstrate GLUT‐1 downregulation, pointing at the crucial role of this mechanism for the anti‐tumor activity of the synthetic rocaglates. Transcriptome profiling revealed glycolysis as one of the major pathways differentially regulated in sensitive and resistant cells. Analysis of synthetic rocaglate efficacy in a 3D tissue context with a co‐culture of tumor and normal cells demonstrated a selective effect on tumor cells and substantiated the mechanistic observations obtained in cancer cell lines. Increased glucose uptake and metabolism is a universal feature across different tumor types. Therefore, targeting this feature by synthetic rocaglates could represent a promising direction for exploitation of rocaglates in novel anti‐tumor therapies.

## Graphical abstract

We demonstrate that novel synthetic rocaglate compounds induce cancer cell death through inhibition of glucose metabolism and glucose uptake mediated by downregulation of glucose transporter‐1 (GLUT‐1). Generation of a rocaglate‐resistant haploid cell line revealed the importance of GLUT‐1 downregulation as a major target for synthetic rocaglates and resistance development. Additionally, the combination with the glucose anti‐metabolite 2DG enhances the anti‐neoplastic activity of synthetic rocaglates, laying the foundation for novel promising rocaglate‐based combination therapy approaches.

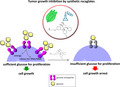

## 1 INTRODUCTION

Rocaglates, also known as flavaglines, are natural compounds sharing common characteristics of their chemical structure.^1^, ^2^ Due to the well‐characterized biological properties, including potent inhibition of translation and therefore strong anti‐neoplastic potential,^3^, ^4^, ^5^, ^6^, ^7^, ^8^, ^9^, ^10^ natural rocaglates and their derivatives raise significant interest as potential drug candidates for tumor treatment. However, the clinical translation of natural rocaglates for cancer treatment has been hampered by costly extraction processes providing only limited amounts of active substances. Recently, the successful chemical synthesis of rocaglates provided a unique opportunity to bring this promising cancer therapeutic agent group into clinical application.^11^ In contrast to natural rocaglates, the synthetic analogues are less well characterized, except for their extensively studied potential in inhibition of translation.^11^, ^12^, ^13^

One of the very common cancer hallmarks is high glucose dependence of cancer cells, also known as Warburg effect.^14^, ^15^ It is known that cancer cells metabolize glucose via aerobic glycolysis, which is energetically less efficient than oxidative phosphorylation. As a result, cancer cells consume substantially more glucose in comparison to normal cells. This principle already found its application in clinics for cancer diagnostics using Positron Emission Tomography, where radioactively labeled 2‐deoxy‐D‐glucose (2DG) is applied to reveal cancer tissues.^16^, ^17^ However, therapeutic agents efficiently targeting glucose metabolism are still lacking.

Although most previous studies reported on the effects of rocaglates related to the inhibition of translation,^11^, ^13^, ^18^, ^19^ emerging evidence suggest involvement of metabolic alterations upon rocaglate treatment in cancer cells.^20^, ^21^ However, the mechanisms of action resulting in metabolic changes in tumor cells remain elusive.

Here, we comprehensively characterize the anti‐neoplastic effects of synthetic rocaglate compounds, with a particular focus on glucose metabolism in cancer cells. We examine the growth‐inhibitory potential of synthetic rocaglate combination with glucose anti‐metabolite 2DG. Moreover, using a haploid cell line with a genetically induced rocaglate‐resistance, we demonstrate the crucial importance of glucose transporter inhibition for the anti‐proliferative efficacy of synthetic rocaglates. Thus, we elucidate a new aspect in the broad spectrum of biological effects of synthetic rocaglates supporting their growth‐inhibitory properties and underlining their high potential as tumor‐targeting substances.

## 2 MATERIALS AND METHODS

### 2.1 Cell lines

HCT116 (RRID:CVCL_0291; ECACC) and HT29 (RRID:CVCL_0320, Tumorbank of the DKFZ Heidelberg) cells were cultured in DMEM supplemented with 10% FBS, 100 U/ml penicillin and 100 µg/ml streptomycin (1% P/S). HGC178 (IMD‐3‐sensitive) and HGC176 (IMD‐3‐resistant) cells were generated from Hap1 cells by Haplogen GmbH (Vienna, Austria) using gene‐trap (GT) mutagenesis and cultured in IMDM + 10% FBS + 1% P/S. Other cell lines were obtained from ECACC and cultured in: DMEM + 10% FBS + 1% P/S (CaSki (RRID:CVCL_1100), HeLa (RRID:CVCL_0030), SiHa (RRID:CVCL_0032)), RPMI1640 + 10% FBS + 1% P/S + 1% MEM‐NEAA (UPCI‐SCC‐90 (RRID:CVCL_1899)). All cell lines were cultured at 37°C with 5% CO_2_. Cell lines were checked for mycoplasma contamination by PCR (Venor^®^ GeM Classic kit). All experiments were performed with mycoplasma‐free cells. Cells were cultured to a maximum of 30 passages after thawing. Cell lines were authenticated using Multiplex Cell Authentication by Multiplexion as described before.^22^ The SNP profiles matched known profiles.

### 2.2 Substances

2DG stock solution was prepared in cell culture medium without supplements. Synthetic rocaglate derivatives (IMD‐1: IMD‐026259, IMD‐3: IMD‐026260) were kindly provided by IMAX Discovery GmbH (Konstanz, Germany). Stock solutions (20 mM) were prepared in DMSO, stored at −20°C and diluted in medium to obtain a 100 µM stock, from which 10x treatment stocks were prepared. If not indicated otherwise, IMD compounds were applied at the end concentration of 100 nM. Cisplatin stock solution (2.5 mM) was prepared in 0.9% NaCl and further diluted in medium.

### 2.3 Cell viability

Cells were seeded onto a 96‐well plate in vertical replicates (n = 3). After 24 h, cells were treated with different drug combinations for 48 h. MTS proliferation assay was performed with the CellTiter 96^®^ AQueous One Solution according to the manufacturer's instruction.

### 2.4 Impedance measurement for real‐time monitoring of cell growth and proliferation

As a label‐free cell density monitoring method, impedance measurement was carried out using the xCELLigence system (E‐Plate VIEW 96‐well, 37°C, 5% CO_2_). Cells were seeded in vertical replicates (n = 3) and space between wells was filled with PBS to reduce evaporation. After 24 h, treatment was started and continued up to two weeks with refreshing the medium (with/without treatment) each 48 h. Impedance values were recorded every 30 min for a total duration of 11 days and the data were analyzed using RTCA 2.0 software. Last measurement before treatment start was used for cell index normalization.

### 2.5 Proliferation

Cells were seeded onto a 96‐well plate (n = 3). After 24 h, cells were treated while simultaneously incubating them with 0.1 µCi/ml [methyl‐^3^H]‐thymidine (2.775 TBq/mmol, Hartmann Analytic GmbH, MT6035) for 48 h. Afterward, cells were washed with medium containing 1 mM non‐labeled thymidine and lysed in 0.5 M NaOH (1 h, RT). Cell lysate was mixed with 10 ml UltimaGold Scintillation Cocktail and subjected to liquid scintillation counting (LSC). Each sample was counted until 2σ was 0.5, this setting was used for all experiments using LSC.

### 2.6 Cytotoxicity

Cells were labeled for 12 h with 10 µCi/ml ^3^H‐thymidine and, after extensive washing to remove not incorporated tracer, seeded onto a 96‐well plate (n = 3). After 24 h, cells were treated for 12, 24, or 48 h with different drug combinations. As a background measurement, untreated cells were harvested before starting the treatment. To harvest the cells, medium was collected and cells were washed with medium containing 1 mM non‐labeled thymidine to stop further incorporation of ^3^H‐thymidine. Medium and wash were collected and mixed with 10 ml UltimaGold. Cells were lysed with 0.5 M NaOH (1 h, RT) and wells were washed. Lysate and wash were mixed with UltimaGold in a new scintillation vial. Medium and lysate were subjected to LSC (Figure S1).

### 2.7 Glucose uptake

Cells were seeded onto 6‐well plates (n = 3) and treated for 24 h before incubation with ^3^H‐labelled 2DG (2‐[1‐^3^H]‐Deoxy‐D‐glucose, 0.74 TBq/mmol, American Radiolabeled Chemicals Inc., 1558). Medium was removed and cells were washed with PBS. Fresh DMEM without supplements containing different glucose concentrations and 0.5 µCi/ml ^3^H‐2DG was added (15 min, 37°C). Afterwards, cells were washed with PBS and trypsinized. After centrifugation (10 min, 290 g), cell pellets were washed with PBS and lysed in 0.2 M NaOH (4°C, overnight). Half of the cell lysate was mixed with 10 ml UltimaGold for LSC. Glucose uptake was normalized to protein (Bradford assay).

### 2.8 Lactate production

Cells were seeded onto 24‐well plates (n = 3) in DMEM containing 1 g/l glucose. After 20 h treatment, the medium was removed and cells were washed once with PBS before adding fresh DMEM (1 g/l glucose), continuing the treatment for 6 h. Medium was collected to measure dissolved lactate. Cells were washed with PBS and lysed (0.2 M NaOH, 4°C, overnight) for protein determination (Bradford assay). After medium processing (see Supplementary Methods), absorption at 339 nm was measured before (A1) and 30 min after (A2) addition of 100 µl L‐LDH. Lactate concentration of the sample was calculated using the Lambert‐Beer law. Each sample was measured in two technical replicates. Lactate concentration was normalized to protein.

### 2.9 Pentose phosphate pathway activity

Parallel to glycolysis, glucose can also be metabolized through pentose phosphate pathway (PPP) generating pentoses including ribose‐5‐phosphate, a precursor molecule for nucleotide synthesis which serve as building blocks for DNA and RNA. To measure glucose flux into PPP, tritium incorporation from ^3^H‐labeled glucose into RNA was analyzed. Cells were seeded onto 6‐well plates (n = 3). Treatment was performed for 24 h while simultaneously incubating with 5 µCi D‐^3^H(U)‐Glucose (1.48 TBq/mmol, American Radiolabeled Chemicals Inc., ART1353). Cells were harvested and RNA extraction was performed using the RNeasy Mini Kit according to the manufacturer's instructions including on‐column DNase I digestion. Afterwards, RNA was mixed with 10 ml UltimaGold for LSC. RNA concentration was determined using a NanoDrop 1000 spectrophotometer for normalization.

### 2.10 Immunofluorescence staining

Cells were grown on BD Falcon^TM^ Culture Slides for 48 h before starting drug treatment for 48 h. Cells were fixed using precooled 80% methanol (4°C, 5 min), before removing the plastic chamber. Dry slides were placed in precooled acetone (−20°C, 2 min) and dried at RT. 4 h prior to fixation, 10 µM nucleoside analogue EdU was added to label proliferating cells. Samples were blocked with 3% BSA solution in PBS (with 0.9 mM CaCl_2_, 0.49 mM MgCl_2_), if required EdU click reaction was performed according to the manufacturer's instructions. Primary antibodies in 3% BSA solution (4°C, overnight): Keratin 19 (Ks19.1, 1:20, Progen Biotechnik GmbH); Cleaved caspase‐3 (Asp175, #9661, 1:100, Cell Signaling Technology); M30 CytoDEATH (12140349001, 1:20, Roche Diagnostics); GLUT‐1 (RB‐9052‐P1, 1:200, Thermo Fisher Scientific), Cytokeratin 14 (NCL‐L‐LL002, 1:100, Leica Biosystems). Samples were washed with PBS with 0.02% Triton X‐100, twice with PBS, and dipped into tab water. Secondary antibodies (Alexa488‐donkey anti‐rabbit IgG, 1:800, Thermo Fisher Scientific; Alexa488‐goat anti‐mouse IgG, 1:800, Thermo Fisher Scientific; Cy3‐donkey anti‐rabbit IgG, 1:1000, Dianova; Cy3‐donkey anti‐mouse IgG, 1:1000, Jackson ImmunoResearch) in 3% BSA solution containing 2 µg/ml DAPI were incubated for 30 min at 37°C followed by 30 min at RT. Described washing steps were performed and slides were mounted with Dako Fluorescent Mounting Medium.

### 2.11 Bradford assay

Protein concentrations were obtained by Bradford assay in a 96‐well plate using Bio‐Rad Protein Assay Dye Reagent Concentrate and BSA solution (20 mg/ml) to obtain a standard curve. Cell lysates were diluted before the assay in either water (RIPA lysates) or 0.2 NaOH. Each sample was measured in 2–3 technical replicates to ensure measurement of one experiment/condition on one plate.

### 2.12 Western blot

Cells treated for 48 h (T75 culture flasks, n = 3) were harvested and pellets were resuspended in 2 ml PBS. 600 µl of each triplicate were pooled for protein isolation, pelleted, and lysed in RIPA buffer containing 1% protease inhibitor. The remaining sample was pelleted and stored for transcriptome analysis. After brief sonication, protein lysates were incubated on ice (30 min), centrifuged (15 min, 4°C, 19,600 g) and concentration was determined (Bradford assay). Western blot analysis was performed as described previously.^23^ Twenty microgram protein were separated on 4–20% polyacrylamide gels and electro‐blotted onto a PVDF membrane. Primary antibodies (4°C, overnight): GLUT‐1 (1:300, Thermo Fisher Scientific), β‐actin (1:20,000, MP Biomedicals), α‐tubulin (1:2,000, Sigma‐Aldrich). Secondary antibodies (1 h, RT): anti‐rabbit IgG (HRP‐linked, 1:2,000, Cell Signaling Technology), anti‐mouse IgG (HRP‐linked, 1:5,000, Cell Signaling Technology). Membranes were stripped using Restore^TM^ Western Blot Stripping Buffer before repeating the procedure to stain actin/tubulin on the same blot.

### 2.13 Statistical analysis

Statistics were performed using Prism software and data are shown as mean ± error, if not stated otherwise. Dataset were analyzed for significance using ANOVA followed by a Tukey's multiple comparison (one‐way) or Bonferroni post‐test (two‐way). *p*‐values smaller 0.05 were considered significant. *p ≤ 0.05; **p ≤ 0.01; *** *p* ≤ 0.001; ns, non‐significant.

### 2.14 Transcriptome analysis

RNA was isolated from the cell pellets. Expression analysis was performed on an Illumina Human HT‐12 v4 chip at the Microarray Unit of the Genomics and Proteomics Core Facility, German Cancer Research Centre (DKFZ). Data analysis of the beadarray expression profiling was carried out following the workflow in the “beadarray” package documentation vignette^24^, ^25^ using R 3.6.1 and Bioconductor 3.9. Detailed data analysis is described in Supplementary Methods. The obtained list of significantly differentially gene (DEG) was used for Ingenuity Pathway Analysis (Qiagen).

### 2.15 Gene‐trap mutagenesis of haploid cells

Generation of IMD‐3‐resistant cells was performed by Haplogen GmbH (Vienna, Austria) using gene‐trap (GT) mutagenesis as described previously^26^ with subsequent resistance screen for IMD‐3. Detailed description is provided in Supplementary Methods.

### 2.16 Organotypic culture model (OTC)

The establishment and cultivation conditions of the organotypic culture model (OTC) mimicking HPV‐induced precancerous lesions are detailed in a methodological paper on this recently developed model that is currently in preparation (Köhler*, Stark* et al.^27^). Technical details on the preparation of the supportive fibroblast‐containing matrix (dermal equivalents) have been described elsewhere.^28^ In these OTCs, SiHa cells and normal keratinocytes were co‐cultivated on dermal equivalents to form tumor cell clusters. After two weeks period of culturing, OTCs received three treatments with 0.1 or 0.5 µM IMD‐3, 1 mM 2DG or the combination of IMD‐3 and 2DG with 48 h intervals and were fixed 7 days after the first treatment and embedded in paraffin for sectioning. Cultures were incubated with 10 µM EdU 4 h prior to fixation.

## 3 RESULTS

### 3.1 Synthetic rocaglates reduce cell viability by inhibiting cell proliferation and inducing apoptosis

**FIGURE 1:**
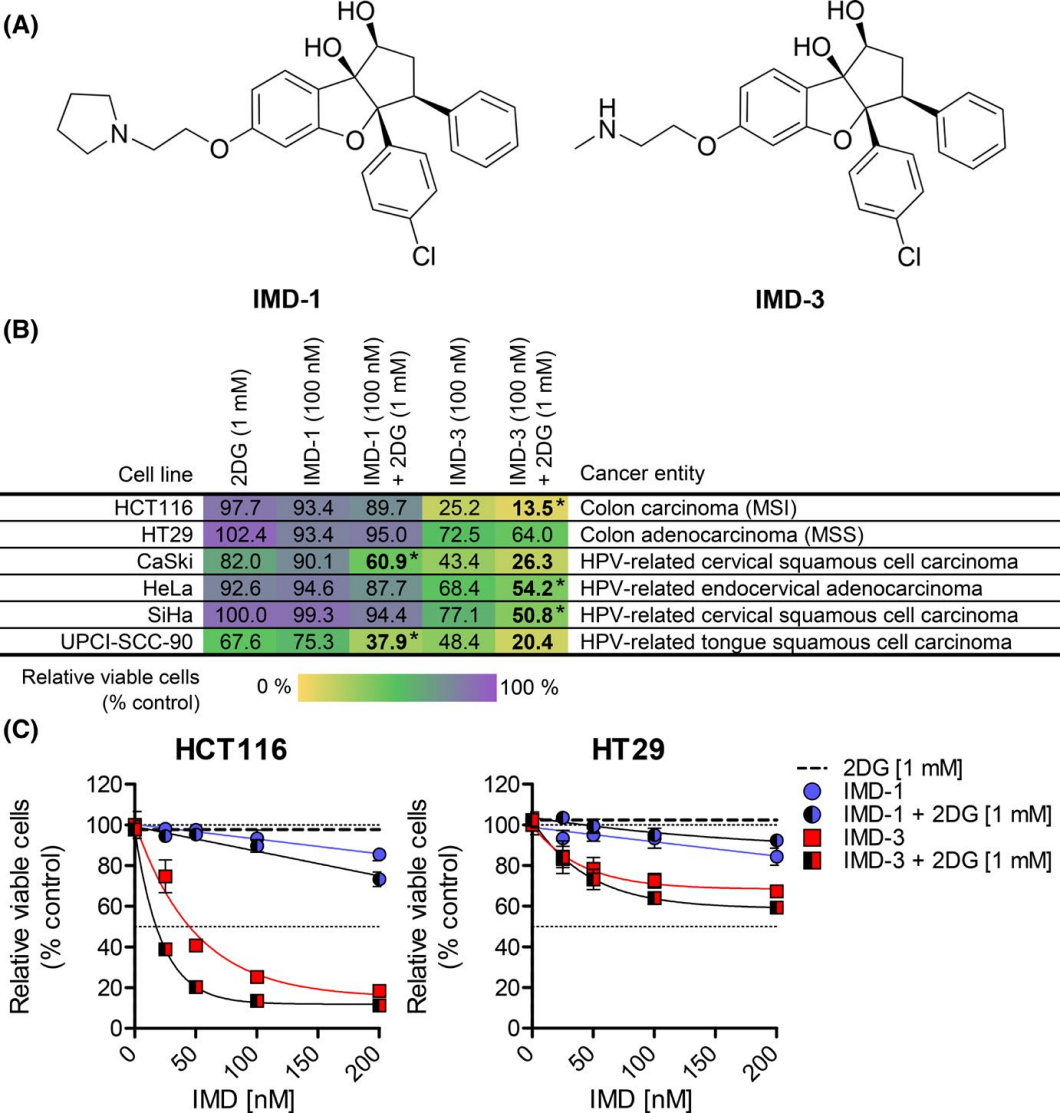
Effects of synthetic rocaglates on cell viability. (A) Chemical structure of synthetic rocaglate derivatives IMD‐1 (IMD‐026259) and IMD‐3 (IMD‐026260). (B) Cell viability (%) upon 48 h treatment with synthetic rocaglates (IMD‐1, IMD‐3) alone and in combination with 2DG. Treatment combinations which were significantly more effective than both single treatments are highlighted in bold letters. Cell lines, in which enhanced growth‐inhibitory effect upon combination of synthetic rocaglates with 2DG was observed, are marked with an asterisk. Significance was determined using two‐way ANOVA and Bonferroni post‐test. Cell lines derived from different tumor tissues including colorectal (HCT116, HT29), cervical (CaSki, HeLa, SiHa), head and neck (UPCI‐SCC‐90). Mean values of relative cell viability are shown. (C) Cell viability upon 48 h treatment with IMD‐1 or IMD‐3 alone and in combination with 2DG (1 mM) in HCT116 and HT29 cells

In order to unravel anti‐cancer properties of synthetic rocaglates, we focused on two lead compounds from the synthetic rocaglate family (IMD‐026259, IMD‐026260; hereafter referred to as IMD‐1 and IMD‐3, respectively, Figure 1A). We first assessed the anti‐tumor characteristics of these two synthetic rocaglate compounds in 22 cancer cell lines derived from different tumor tissues including colorectal, cervical, prostate, breast cancer and others (Figure 1A,B, S2A). Both substances were applied alone and in combination with 2DG (1 mM), a potent inhibitor of glucose metabolism.^29^, ^30^, ^31^ The most potent reduction of cell viability upon a single compound treatment was observed with IMD‐3, with an average IC_50_ of 73 nM in 13 cell lines, which could be further reduced to an average of 51 nM in 17 cell lines when combined with 2DG (Figure S2B). IMD‐1 and 2DG alone did not strongly reduce cell viability in most of the tested cell lines. However, the combination of IMD‐1 with 2DG reduced IC_50_ of the former (Figure S2B) and exhibited an enhanced growth inhibitory effect in several cell lines (e.g., HCT116, HeLa, SiHa). To further characterize the effects of synthetic rocaglates, we focused on two colon carcinoma cell lines: a more treatment‐sensitive microsatellite‐unstable HCT116 (IC_50_ of IMD‐3: 50 nM) and a less sensitive microsatellite‐stable HT29 (IC_50_ not reached) (Figure 1C) and validated our major findings in HPV‐induced cervical cancer cells lines (SiHa, CaSki, HeLa, HNSCC UPCI‐SCC‐90).

**FIGURE 2:**
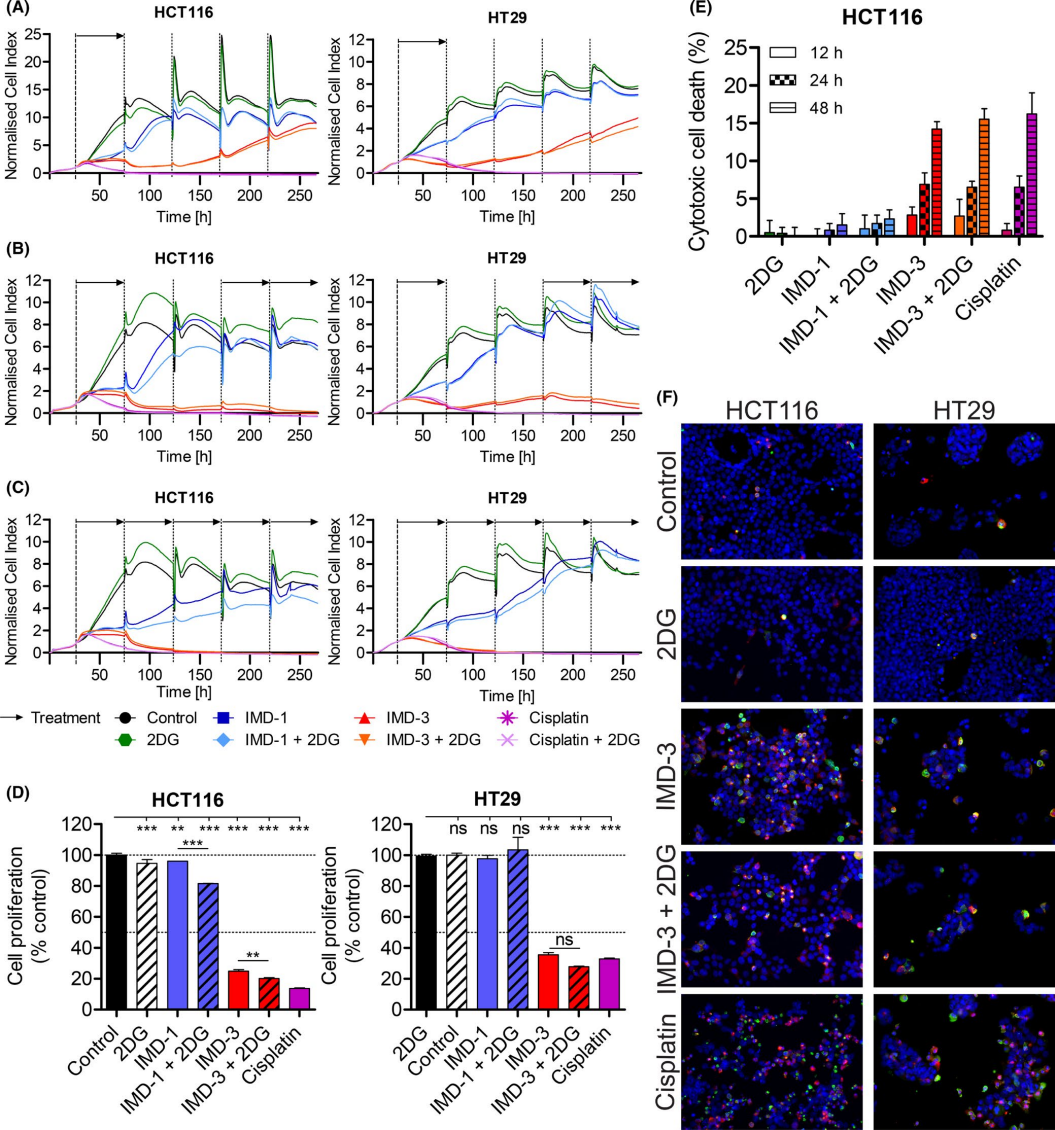
Inhibition of cell proliferation and apoptosis induction in HCT116 and HT29 cells upon treatment. (A) Real‐time proliferation monitoring of cells treated for 48 h before changing back to drug‐free medium. The peaks of the cell index result from changing the medium, and fluctuations of the cell index result from the overconfluence of the cells treated with vehicle or less efficient substances. (B) Real‐time proliferation monitoring of cells treated for 48 h before interrupting the treatment for 96 h followed by treatment re‐application. (C) Real‐time proliferation monitoring of cells continuously treated for 11 days . Mean values of cell proliferation are shown for cell index values to increase readability of the data. (D) Proliferation inhibition during 48 h of treatment measured by ^3^H‐thymidine incorporation. (E) Cytotoxic cell death induced by treatment in HCT116 cells based on the release of ^3^H‐thymidine. Spontaneous cell death (release observed in control cells) was subtracted to obtain cytotoxic cell death of each substance (Figure S3). (F) Immunofluorescence staining for apoptosis using cleaved caspase‐3 (red) and caspase‐cleaved cytokeratin 18 (green). Nuclei were DAPI‐stained. Magnification: 400‐fold. 2DG: 1mM, IMD‐1: 100 nM, IMD‐3: 100 nM, Cisplatin: 75 µM. *p ≤ 0.05; **p ≤ 0.01; ***p ≤ 0.001; ns, non‐significant

To characterize the influence of synthetic rocaglates on cell proliferation, cell density, and adhesion in real‐time, cells were monitored over time by impedance measurement (Figure 2). In comparison to steeply increasing growth curves of cells treated with vehicle or 2DG, the descending growth curves of IMD‐3‐treated cells reflecting reduction of the impedance values indicated proliferation inhibition. When IMD‐3 treatment was discontinued, the effect was preserved for 48–72 h after treatment interruption and could be restored upon treatment re‐application (Figure 2A,B). In contrast, IMD‐1 even upon continuous treatment merely caused a delay in cell outgrowth, but did not substantially influence the cell density after 120 h (Figure 2), whereas continuous treatment with IMD‐3 prevented the HCT116 and HT29 cells from re‐growing (Figure 2C). Next, we asked what main mechanisms are responsible for cancer cell growth inhibition upon treatment with synthetic rocaglates. The ^3^H‐thymidine incorporation assay showed that the large proportion of the induced effect was caused by proliferation inhibition, whereas only a minor effect could be attributed to direct cytotoxicity in the treated cells (Figure 2D,E, S3). Proliferation inhibition was confirmed in HPV‐transformed cervical cancer cells (Figure S4). In addition, cells treated with IMD‐3 showed phenotypic hallmarks of apoptosis, such as expression of cleaved caspase‐3 and caspase‐cleaved cytokeratin 18 (Figure 2F).

### 3.2 Synthetic rocaglates inhibit glucose metabolism through reduction of GLUT‐1 expression

**FIGURE 3:**
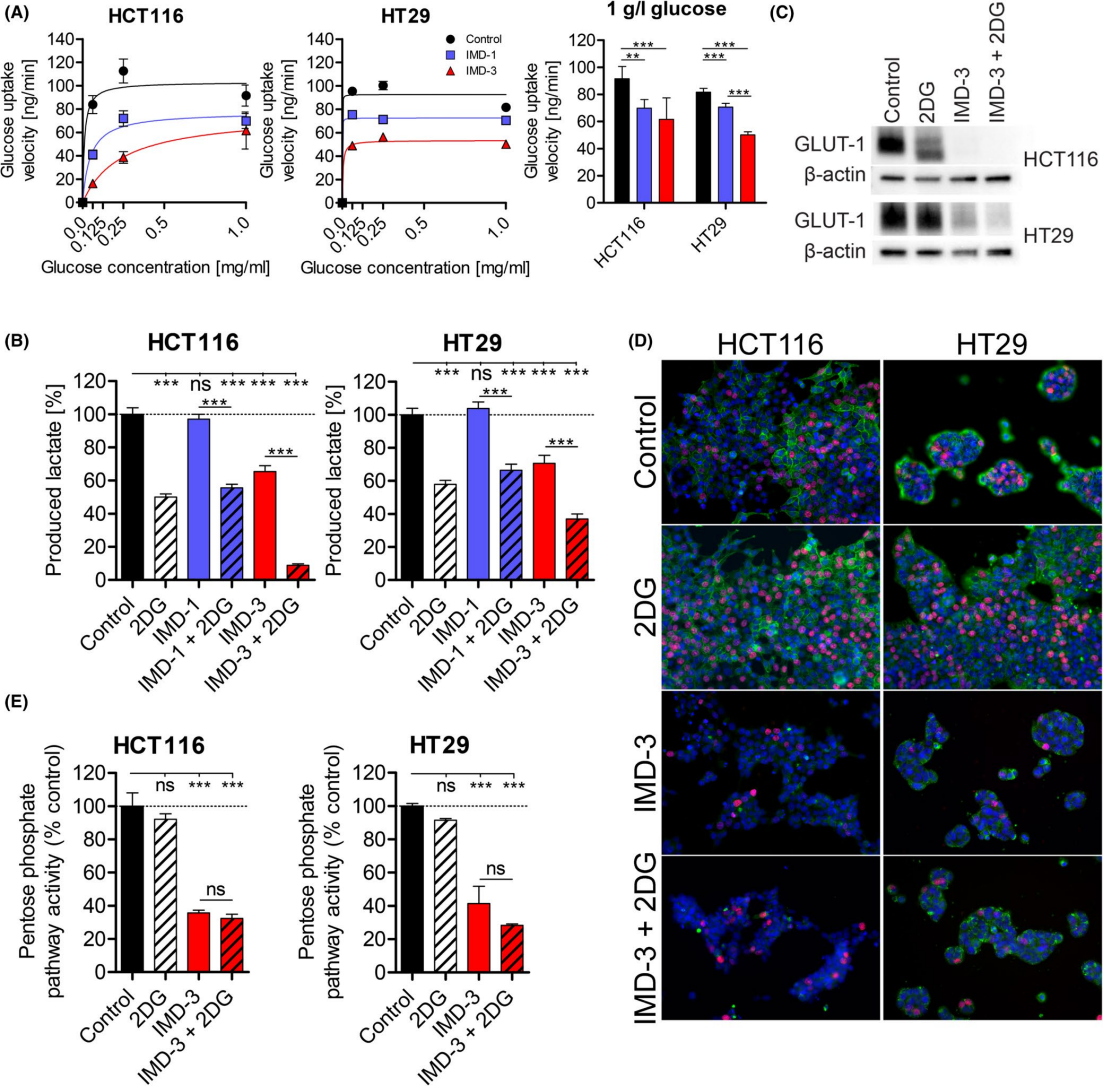
Influence of synthetic rocaglates on glucose metabolism. (A) Glucose uptake velocity upon synthetic rocaglate treatment (24 h) dependent on the glucose concentration in the medium using radioactive ^3^H‐2DG. (B) Amount of secreted lactate by IMD‐treated cells after 24 h of treatment. Inhibition of glycolysis shown by reduced amount of secreted lactate upon 2DG treatment can be observed in both cell lines, whereas of both rocaglates only IMD‐3 is able to inhibit glycolysis. (C) Western blot analysis of GLUT‐1 (45–55 kDa) showing decreased (HT29) to abolished (HCT116) expression upon IMD‐3 treatment. (D) Immunofluorescent staining of glucose transporter GLUT‐1 (green) and the proliferation marker EdU (red) showing reduced GLUT‐1 expression and proliferation inhibition by IMD‐3 treatment of cancer cells. Nuclei were DAPI stained. Magnification: 400‐fold. (E) PPP activity measured by radioactivity of ^3^H added to the cells in form of ^3^H‐labeled glucose and later incorporated into RNA. *p ≤ 0.05; **p ≤ 0.01; ***p ≤ 0.001; ns, non‐significant. 2DG: 1 mM, IMD‐1: 100 nM, IMD‐3: 100 nM

Previously, natural rocaglates were suggested to influence glucose flux into cancer cells.^20^ As we observed an enhanced growth‐inhibitory effect of IMD‐3 when combined with 2DG, we asked, whether glucose metabolism is influenced also by synthetic rocaglates. We first measured glucose uptake upon IMD treatment using radioactive ^3^H‐2DG as tracer and found that treatment with synthetic rocaglates reduced glucose uptake velocity (Figure 3A, S4C). In line with a more potent effect of IMD‐3 compared to IMD‐1 on cell viability, glucose uptake was more strongly inhibited in IMD‐3‐treated cells. Consequently to reduction of glucose uptake, the glycolytic activity of cells was more substantially reduced upon treatment with IMD‐3. Importantly, the combination of IMD‐3 and 2DG almost completely abolished the glycolytic activity in treated cells (Figure 3B, S4D).

To examine the possible mechanisms, through which glucose uptake is deregulated in cancer cells upon treatment, we investigated the expression of the most abundant glucose transporter GLUT‐1, known to be overexpressed in the majority of tumors.^32^, ^33^, ^34^ Strikingly, cellular GLUT‐1 protein expression was significantly downregulated or completely eliminated upon IMD‐3 treatment, whereas 2DG treatment did not alter the expression of GLUT‐1 (Figure 3C), which fits to the hexokinase inhibition‐based anti‐glycolytic function of 2DG. This finding was validated by the immunofluorescent staining of treated cells revealing loss of membranous GLUT‐1 expression upon IMD‐3 treatment (Figure 3D, S4B).

As glucose metabolism links together catabolic and anabolic pathways in cells, it is important to analyze possible treatment‐related re‐direction of glucose from energy‐producing into nucleic acid building blocks‐generating pathways, such as pentose phosphate pathway (PPP). However, the incorporation of glucose chemical derivatives into nucleic acids was not increased but decreased upon treatment with IMD‐3, indicating no shift of glucose metabolism toward the anabolic pathways upon IMD‐3‐related glycolysis inhibition, and reflecting rather the generally decreased glucose flux (Figure 3E). Lack of required precursor molecules such as ribose‐5‐phosphate for nucleotide and nucleic acid synthesis also explains the observed proliferation inhibition upon synthetic rocaglate treatment.

In general, both cell lines demonstrated inhibition of glucose metabolism; consistent with the higher treatment sensitivity of HCT116 cells compared to HT29, the effects of IMD‐3 on glucose metabolism were more pronounced in HCT116 cells.

### 3.3 Gene‐trapped treatment‐resistant cells do not exhibit deregulation of glucose metabolism

**FIGURE 4:**
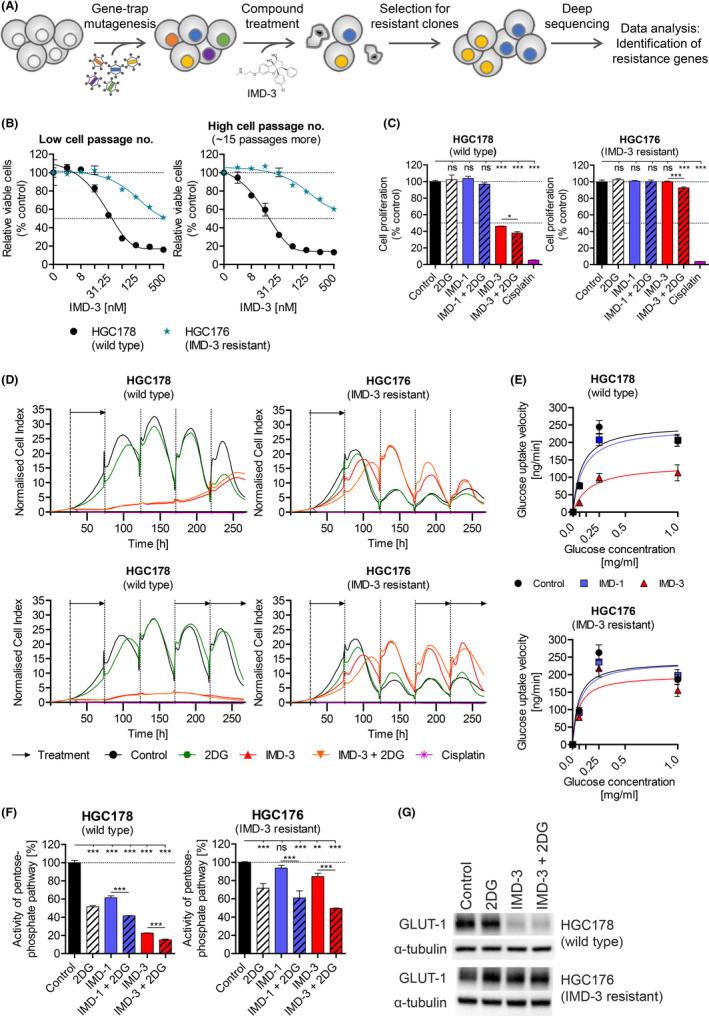
Influence of synthetic rocaglates on glucose metabolism in IMD‐3‐resistant cells. (A) Schematic description of IMD‐3‐resistant cell line generation. (B) Cell viability upon 48 h IMD‐3 treatment in IMD‐3‐ sensitive (HGC178) and resistant (HGC176) cells. HGC176 cells remain resistant over repeated passaging. (C) Cell proliferation during 48 h of treatment measured by ^3^H‐thymidine incorporation. (D) Impedance measurement over 11 days either treating the cells for only 48 h before changing back to medium without treatment (upper panel) or interrupting the treatment for 4 days (lower panel). The peaks of the cell index result from changing the medium, and fluctuations of the cell index result from the overconfluence of the cells treated with less efficient substances. Mean values of cell proliferation are shown for cell index values to increase readability of the data. (E) Glucose uptake velocity upon synthetic rocaglate treatment dependent on the glucose concentration in the medium. IMD‐3 reduced the uptake velocity at 1 g/l glucose in both cell lines, however the effect was significantly stronger in sensitive HGC178 cells compared to resistant HGC176 cells (Figure S5B). (F) PPP activity in HGC178 (sensitive) and HGC176 (resistant) cell lines upon treatment with rocaglates and 2DG. (G) Western blot analysis of GLUT‐1 expression upon IMD‐3 treatment (alone or with 2DG) in HGC178 cells showing close to abolished expression, whereas HGC176 cells retained GLUT‐1 expression upon treatment. *p ≤ 0.05; **p ≤ 0.01; ***p ≤ 0.001; ns, non‐significant. 2DG: 1mM, IMD‐1: 100 nM, IMD‐3: 100 nM, if not indicated otherwise; Cisplatin: 75 µM

To elucidate possible genetic mechanisms behind the efficacy of synthetic rocaglates, especially IMD‐3, a resistant haploid cell line was generated using gene‐trap (GT) mutagenesis for high‐throughput genetic resistance screening (Figure 4A). For gene‐trap mutagenesis, upon random insertion of a cassette consisting of splice acceptor, reporter gene, a termination sequence and short unique DNA sequence (barcode) into intronic gene regions of treated cells, a fusion transcript is generated leading to an impaired expression of the trapped gene.^26^ Next, if any cells became resistant to treatment upon mutagenesis, massive parallel sequencing (NGS) was applied to identify the trapped gene possibly responsible for resistance formation. Using this approach, we were able to obtain cells that became resistant to IMD‐3 treatment after GT mutagenesis, which were further cultured and established as a resistant cell line (HGC176) from an original wild type (WT) cell line HGC178.

To check, whether the induced resistance against IMD‐3 treatment is merely transient or remains stable over time, both WT HGC178 and resistant HGC176 cells were cultured over a period of more than 5 weeks and treatment sensitivity was compared between cells from low and high passages. Importantly, the HGC176 cell line showed stable resistance towards IMD‐3 as demonstrated by consistent cell viability in growing cell passages (Figure 4B). In sharp contrast to WT HGC178 cells, proliferation in resistant HGC176 cells was not influenced by IMD‐3 treatment (Figure 4C). Treatment with 2DG alone also had no pronounced effect on cell proliferation in both cell lines, whereas combination of 2DG with IMD‐3 showed a slightly enhanced anti‐proliferative effect in both cell lines. Moreover, real‐time proliferation monitoring by impedance assay confirmed the observed stable resistance of HGC176 cells against IMD‐3 in long‐term (11 days) experiments with different treatment schemes (Figure 4D). Here, HGC176 cells retained resistance also after interruption and re‐application of treatment with IMD‐3, whereas HGC178 cells remained sensitive (Figure 4D, lower panel). However, the sequencing analysis did not identify a gene responsible for resistance, pointing toward a genetics‐independent resistance mechanism sustaining IMD‐3 resistance during continuous passaging in the absence of IMD‐3 (Figure S5A).

We then analyzed whether IMD‐3‐resistant cells show glucose metabolism alterations described above in the HCT116 and HT29 cell lines. The comparison between WT HGC178 and resistant HGC176 cell lines revealed substantially less pronounced reduction of glucose uptake upon treatment in HGC176 (Figure 4E, S5B). Also, in contrast to dramatic reduction of glucose flux into PPP pathway in the WT cell line, the effect of IMD‐3 treatment was significantly less pronounced in the resistant HGC176 cell line (*p* ≤ 0.001) compared to the WT cells (Figure 4F). Noteworthy, the effect of 2DG on PPP in resistant cells was weaker compared to sensitive cells (*p* ≤ 0.001). Importantly, protein levels of GLUT‐1 remain unaffected in HGC176 cells upon IMD‐3 treatment (Figure 4G). Taken together, these observations point out the important role of glucose metabolism inhibition and GLUT‐1 downregulation in the anti‐tumor efficacy of IMD‐3.

### 3.4 Treatment with synthetic rocaglates leads to alteration of metabolic pathway genes

As the gene sequencing did not reveal a certain genetic alteration possibly responsible for resistance development, we performed RNA transcriptome analysis to detect altered pathways.

**FIGURE 5:**
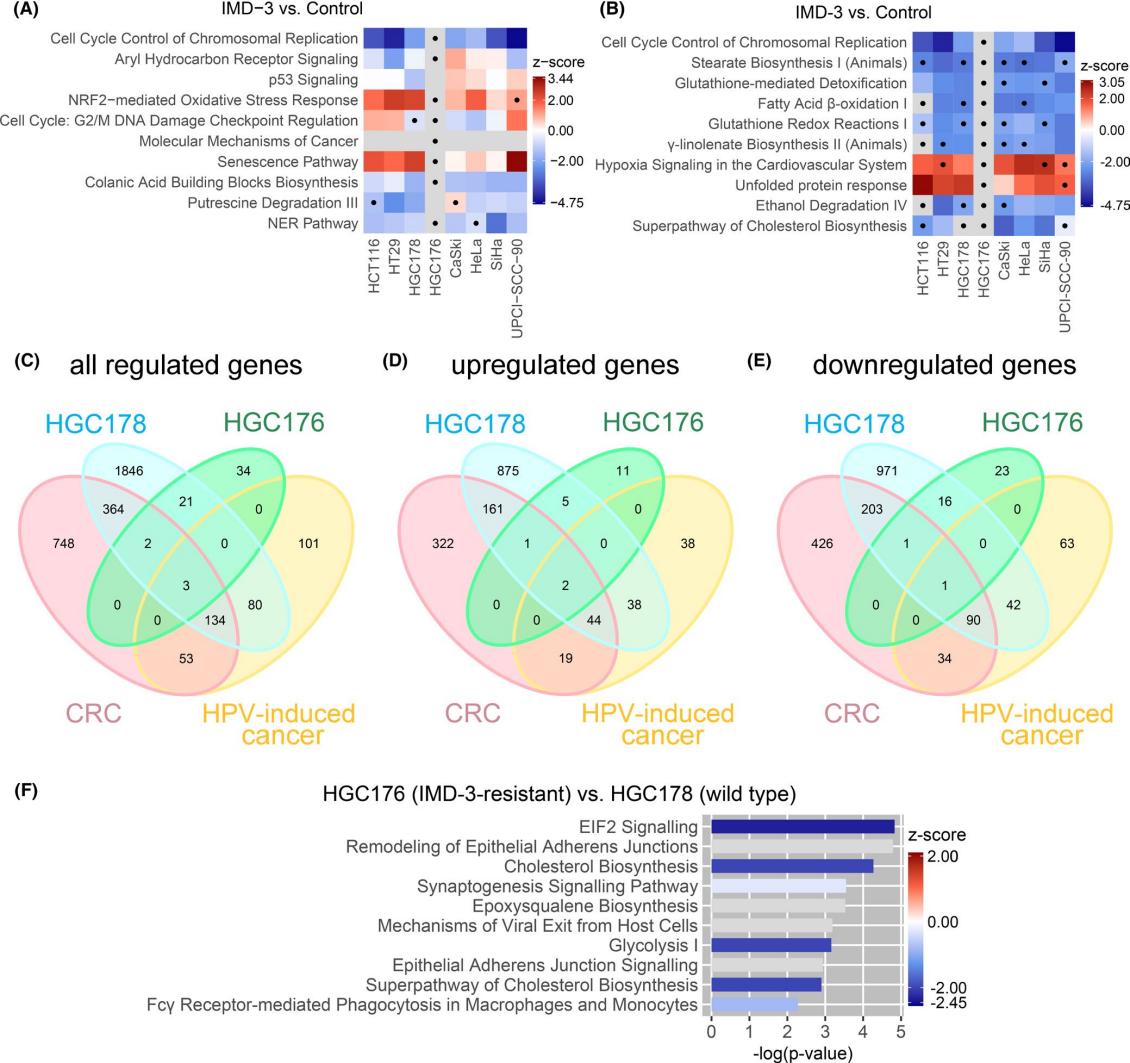
Analysis of RNA transcriptome data. (A) Top 10 deregulated canonical pathways (IPA) in IMD‐3 treated cells (100 nM, 48 h) sorted by their p‐value. Displayed are the z‐scores for each pathway predicting the activation (z‐score ≥ 2; red) or inhibition (z‐score ≤ −2; blue) of the pathways based on the transcriptome data. If no direction of the pathway could be predicted: z‐score = 0 (white). If the z‐score could not be calculated, pathways are labelled gray. Pathways with *p*‐values > 0.05 are marked with a dot. (B) Top 10 deregulated canonical pathways in IMD‐3‐treated cells (100 nM, 48 h) sorted by their z‐score. (C) All shared significantly DEGs upon IMD‐3 treatment considering only genes which were regulated in the same direction (up‐ or downregulated) in the compared cell lines. ‘CRC’ summarizes genes shared between HCT116 and HT29 cells. `HPV‐induced cancer’ summarizes the HPV‐transformed cervical cell lines CaSki, HeLa, SiHa and HNSCC UPCI‐SCC‐90. (D) Only shared significantly DEGs which were upregulated in compared cell lines. (E) Only shared significantly DEGs which were downregulated in compared cell lines. (F) Top 10 deregulated canonical pathways (IPA) between IMD‐3‐resistant HGC176 and IMD‐3‐sensitive HGC178 cells. Transcriptome data are listed in Table S1.

Pathway analysis revealed that IMD‐3 treatment influenced similar pathways in all treatment‐sensitive cell lines (HCT116, HT29, HGC178, CaSki, HeLa, SiHa, UPCI‐SCC‐90), whereas those pathways were not deregulated in IMD‐3‐resistant HGC176 cells (Figure 5A, B). Similar to natural rocaglates, which were found to promote ER stress leading to apoptosis,^35^, ^36^ IMD‐3 strongly induced “unfolded protein response” pathway indicating ER stress (Figure 5B), confirming our observation of IMD‐3‐induced apoptosis. Additionally NRF2‐mediated oxidative stress response was upregulated upon IMD‐3 treatment, whereas glutathione (GSH) pathways were downregulated (Figure 5A,B), indicating a depletion of GSH and accumulation of ROS in sensitive cells, which has been previously found to induce apoptosis.^37^, ^38^ GSH depletion could be mediated by inhibition of the PPP upon synthetic rocaglate treatment decreasing cellular NADPH levels, thus hampering the activity of glutathione reductase enzyme, which catalyzes the reduction of glutathione disulfite (GSSG) to GSH.

Heat shock Factor 1 (HSF1) is a master regulator of transcriptional programs linking the translational flux to the metabolic state of the cells and enabling the adaptation to the high anabolic demands of cancer cells.^20^ Interestingly, IMD‐3 treatment resulted in decreased levels of the heat shock protein family member HSPA8 (4‐fold) in HCT116 cells, indicating translation inhibition, whereas HSPA8 was increased in resistant HGC176 cells upon treatment (2.8‐fold), representing the most upregulated gene. At the same time, HT29 showed a 10‐fold increase in expression levels of TXNIP, a negative regulator of glucose flux,^39^ upon treatment, which was also increased in all four HPV‐transformed cell lines. Furthermore, observed downregulation of GLUT‐1 on protein level was also detected in HPV‐transformed CaSki cells on the transcriptome level (2.3‐fold, Table S1). Transcriptome analysis further showed inhibition of oxidative phosphorylation by downregulation of electron transport chain genes through IMD‐3 treatment, especially downregulation of the complex I assembly factor NDUFAF3, or complex II subunit SDHC (Table S1). Comparison of all significantly differentially expressed genes (DEGs) upon IMD‐3 treatment between cell lines revealed that 137 genes were regulated in the same direction in all tested treatment‐sensitive cell lines, while only three of these genes were also regulated in IMD‐3‐resistant HGC176 cells (Figure 5C–E). DDIT3 was upregulated up to 20‐fold (HCT116) in all treatment‐sensitive cell lines, which might indicate activation of the integrated stress response upon IMD‐3 treatment.^40^, ^41^

Due to the striking difference between IMD‐3‐sensitive and resistant cells on transcriptome level upon IMD‐3 treatment, we investigated the transcriptomic difference between untreated WT HGC178 and resistant HGC176 cells (Figure 5C). The analysis identified five pathways downregulated in the resistant cells compared to WT cells, two of which were "eIF2 signaling", responsible for translation initiation, and "glycolysis", both shown to be possible targets of natural and synthetic rocaglates (Figure 5C). The downregulation of glycolysis in resistant cells was further substantiated by their decreased sensitivity towards 2DG treatment (Figure S5C). Additionally, IMD‐3‐resistant cells showed downregulation of oxidative phosphorylation in comparison to WT cells. Interestingly, resistant HGC176 cells showed a 7.5‐fold downregulation of HSPA8 in comparison to WT HGC178 cells, although HSF1 levels remained unaffected (Table S1). These changes might indicate that HGC176 acquired their resistance to IMD‐3 through transcriptional alterations rather than mutations of single genes leading to adaptations of the cellular metabolism, which remain stable over continuous passaging sustaining acquired IMD‐3 resistance.

### 3.5 Synthetic rocaglates reduce growth of tumor cell clusters in a 3D co‐culture model

**FIGURE 6:**
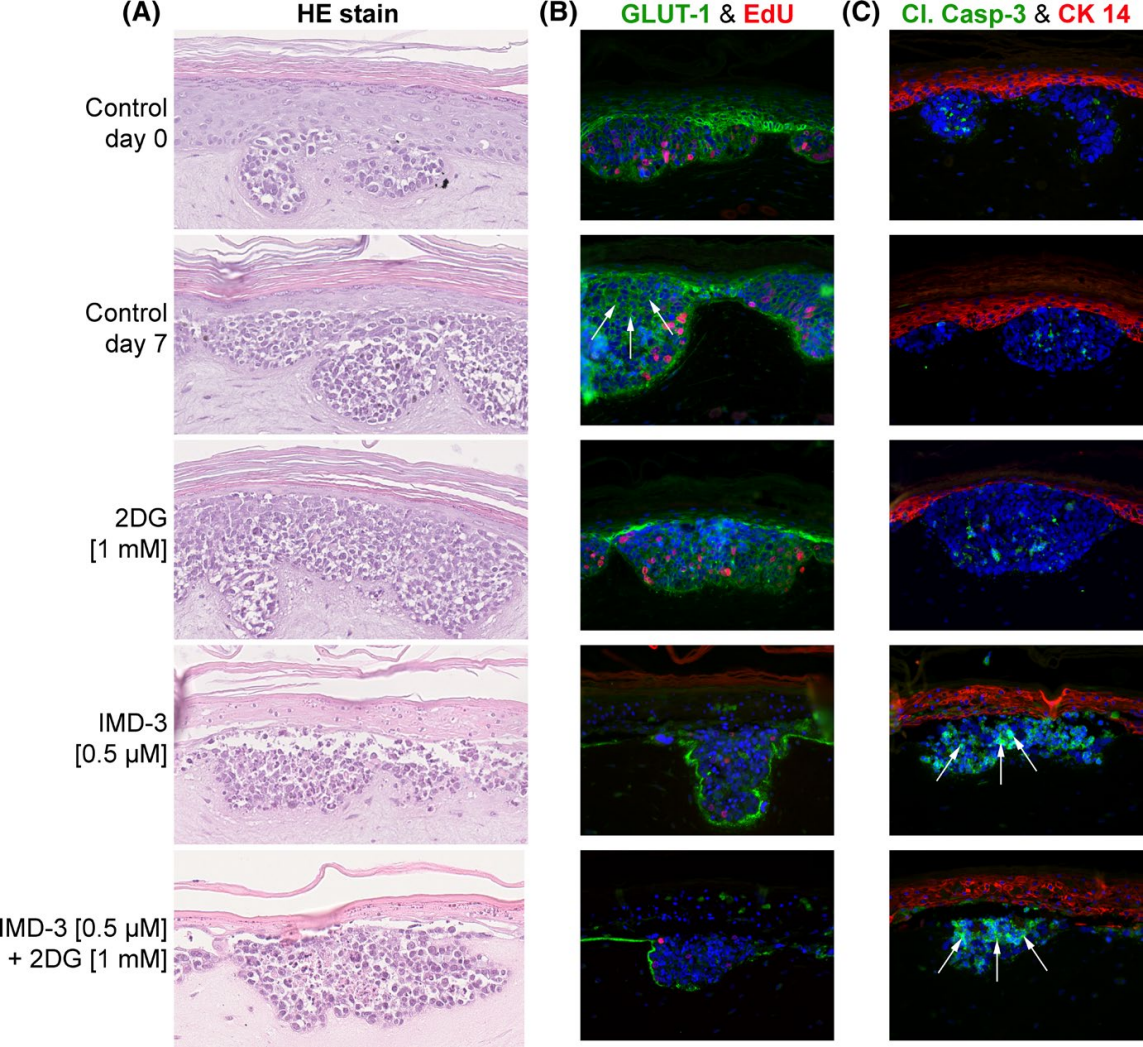
Growth of tumor cell clusters in OTCs of SiHa tumor cells and normal keratinocytes under treatment with 2DG and synthetic rocaglates. (A) Decreased growth and viability of tumor cell clusters in treated OTCs visualized in HE staining. (B) Fluorescence staining of OTCs showing reduced glucose transporter GLUT‐1 (green) levels and decreased proliferation (EdU, red). (C) Fluorescence staining for cleaved caspase‐3 (green) indicates apoptosis induction. Keratinocytes are stained for cytokeratin 14 (red). Nuclei are stained with DAPI (blue).

In order to measure the effect of the treatment on tumor and non‐tumorous cells in parallel, we used an organotypic co‐culture (OTC) model of normal keratinocytes and HPV‐induced cervical tumor cells recently established in our group.^27^ In contrast to control OTCs treated with vehicle or 2DG showing growth of tumor cell clusters during the entire experiment period, OTCs treated with IMD‐3 and the combination of IMD‐3 and 2DG showed decreased growth of tumor cell clusters (Figure 6A). In contrast to 2D culture, treatment with 100 nM IMD‐3 did not exert a substantial effect neither on normal nor on tumor cells (Figure S6), whereas treatment with 500 nM IMD‐3 resulted in reduced the viability of tumor cell clusters in the 3D model. Cell death induction upon IMD‐3 treatment caused nuclear granularization and disruption of the cellular structure in tumor cell clusters (Figure 6).

Next, we analyzed whether the mechanisms of action of IMD‐3 unraveled by experiments in 2D culture could be reproduced in a 3D context with OTCs. To that end, proliferation, apoptosis and GLUT‐1 expression was analyzed upon treatment of OTCs. This analysis confirmed the inhibition of proliferation and induction of apoptosis in tumors cells upon IMD‐3 treatment (Figure 6B,C) previously also observed in monolayer cultures. Importantly, comparable effects on normal keratinocytes were not observed under any of the treatment conditions. Furthermore, the expression of GLUT‐1 was visibly decreased in OTCs treated with IMD‐3 alone or in combination with 2DG, further substantiating the role of GLUT‐1 as one of the major targets of synthetic rocaglates (Figure 6B). Taken together, these data support the results obtained in 2D cell culture and provide first insights into the potential mode of action of synthetic rocaglates in a 3D tissue culture context.

## 4 DISCUSSION

In the present study, we demonstrate inhibition of glucose metabolism as a key biological mechanism of action of synthetic rocaglates, particularly of IMD‐3 (IMD‐026260). We found that IMD‐3 restricts glucose uptake through inhibition of glucose transporter GLUT‐1 expression, leading to hampered glycolysis, apoptosis induction and inhibition of cell proliferation.

Deregulation of cellular metabolism displays a common hallmark for different tumor types fueling tumor cell proliferation.^14^, ^15^, ^42^ To match their increased glucose requirements, cancer cells increase their glucose uptake through upregulation of glucose transporters, especially GLUT‐1 with a high affinity for glucose.^43^ This mechanism had direct clinical implications: for instance, tamoxifen treatment in breast cancer patients was shown to downregulate glycolysis and GLUT‐1 expression,^44^ whereas high expression of GLUT‐1 was linked to poor prognosis in patients with oral squamous cell carcinomas.^33^ Thus, GLUT‐1 may represent a promising protein for targeting high glucose consumption of cancer cells.

By employing GT mutagenesis we generated a cell line stably resistant against treatment with IMD‐3, representing a valuable model for studying mechanisms of action of synthetic rocaglates. Using this newly generated GT cell line, we were able to demonstrate downregulation of GLUT‐1 as a crucial biological effect of IMD‐3 treatment, as in sharp contrast to the tested sensitive cell lines, the IMD‐3‐resistant cell line did not show GLUT‐1 downregulation upon treatment. In fact, RNA transcriptome data supported these results revealing an a priori downregulation of glycolysis in IMD‐3‐resistant cells, further substantiated by decreased sensitivity of IMD‐3‐resistant cells towards 2DG. Thus, downregulated glycolysis prevented further inhibition of glucose metabolism by treatment, suggesting a predictive role of the enhanced baseline glucose turnover and GLUT‐1 expression for the IMD‐3 treatment success. Importantly, treatment‐related reduction in glycolytic activity in the sensitive cell lines did not result in the redirection of the available glucose into pentose phosphate pathway, further suggesting the general restriction of glucose availability for diverse downstream pathways upon treatment.

Together with glycolysis, translation initiation, previously shown to be strongly hampered by natural and synthetic rocaglates,^11^, ^13^, ^18^, ^19^ was among the top five pathways downregulated in the resistant cell line compared to the sensitive WT cell line. These observations are in line with previous reports demonstrating a connection between translation inhibition, a well‐studied feature of natural and synthetic rocaglates, and glucose metabolism.^20^, ^21^ Santagata et al. first reported inhibition of glucose flux in cancer cells upon translation inhibition induced by treatment with rocaglates, which was mediated by inactivation of HSF1 and subsequent upregulation of TXNIP, one of the main negative regulators of glucose uptake.^20^ Our transcriptome data showing decreased expression of HSPA8 and increased expression of TXNIP upon treatment with synthetic rocaglates further support the previously suggested fundamental link between anabolic and catabolic processes in cancer cells. Besides GLUT‐1 downregulation demonstrated on protein and mRNA levels, other glucose transporters such as GLUT‐5 (in CaSki, HeLa) and GLUT‐6 (in HCT116, HeLa), which are less expressed in these tumors and therefore presumably are less relevant for glucose uptake in these tissues also showed downregulation upon IMD‐3 treatment on transcriptome level.^43^ GLUT‐6 has also been previously shown to be downregulated upon treatment of pancreatic tumor tissues with another synthetic rocaglate compound.^21^ These observations underline a general character of synthetic rocaglate‐induced glucose transporter inhibition, which is likely to be most pronounced in the abundantly represented transporter molecules of the respective tissue. Taken together, these findings show that glycolysis and translation initiation are the major target pathways of synthetic rocaglates, although direct interaction of synthetic rocaglates with a specific target molecule remains to be discovered.

In addition to inhibition of translation initiation and glycolysis, we found downregulation of GSH‐related pathways and upregulation of NRF2‐mediated oxidative stress response pathway upon IMD‐3 treatment, leading to apoptosis. Similar findings were reported in a recent study by Chan et al. focusing on another synthetic rocaglate CR‐31,^21^ suggesting common traits in the mechanisms of action of synthetic rocaglates with different chemical structure. Cancer evolution is based on the survival of the fittest clone, thus multiple adaptation tools are exploited by cancer cells in order to survive under certain conditions. Thus, it is not surprising that under IMD‐3 treatment, which deprives cells of glucose, mRNAs levels of genes involved in glutamine metabolism, which serves as an alternative fuel, are increased, presumably, shifting cellular metabolism towards glutamine consumption. In line with this assumption, combination of synthetic rocaglates with glutaminase inhibitor previously showed promising results,^21^ although the influence of IMD‐3 on glutamine metabolism and potential combination with glutaminase inhibitors needs to be further investigated in future studies. Furthermore, pathway analysis revealed alterations of other metabolic pathways upon IMD‐3 treatment, such as fatty acid metabolism, which shall be further investigated in the future to elucidate the general influence of synthetic rocaglates on cellular metabolism.

Our study has strengths and limitations. Major strength is demonstration of the importance of glucose metabolism for the successful treatment with IMD‐3 using a therapy‐resistant *in vitro* cancer model, leading to emergence of possible therapy‐relevant predictive markers, such as GLUT‐1. Moreover, we for the first time demonstrate that the effect of synthetic rocaglates can be enhanced by combination with 2DG, a well‐studied substance tested in several clinical trials, which so far could not be admitted for clinical application due to limited anti‐tumor effect at non‐toxic doses.^29^, ^45^ We also validated our mechanistic observations in an independent set of cancer cells lines from a different cancer entity, further pointing at the generalizable character of the reported findings for several molecularly and clinically distinct tumors. Furthermore, we could demonstrate the efficacy of synthetic rocaglates in a 3D co‐culture model. This model revealed a selective effect of IMD‐3 on the tumor cells and confirmed the mechanistic characteristics of the treatment observed in 2D culture, such as downregulation of GLUT‐1 expression and reduction of cell growth and viability via inhibition of proliferation and induction of apoptosis. The different responsiveness of tumor cells and normal cells indicates a potential therapeutic window for synthetic rocaglates. In addition to pointing at the selective character of the treatment, decreased sensitivity of keratinocytes might result from the decreased metabolic rate in skin^46^ rendering them less sensitive towards the influence of synthetic rocaglates on cellular metabolism. Therefore, 3D tumor models including other normal cells should be used to further validate the selective anti‐tumor effect of synthetic rocaglates. Such studies also need to address the mechanisms responsible for differences in effective drug concentrations between 2D and 3D tumor models, which may be related to impeded diffusion and decreasing drug concentration gradient towards tumor cells occurring in 3D, but not 2D models. Moreover, *in vivo* studies evaluating the treatment parameters not addressable in 2D and 3D tumor models are warranted for further evaluating the potential of synthetic rocaglates as anti‐tumor compounds. Analysis of only two synthetic rocaglates compounds is one limitation of our study. In addition, the *in vivo* efficacy of the synthetic rocaglate treatment should be analyzed in future studies, also investigating the potential effect of combination with 2DG, which could possibly pave the way towards clinical translation of both compounds.

In summary, our study shows that compounds targeting glucose uptake and inhibiting glucose metabolism in cancer cells represent a promising direction for development of cancer‐therapeutic approaches and suggests synthetic rocaglates, particularly IMD‐3, as candidate compounds with strong anti‐cancer potential.

## CONFLICTS OF INTEREST

E.S.P. has received lecture fees by MSD SHARP & DOHME GmbH and Institut für Frauengesundheit (IFG) GmbH. E.S.P. is involved in a research project funded by MSD SHARP & DOHME GmbH outside the submitted work.

## Supporting information

Supplementary Material

Table S1
