# Supplementary material for: Treatment resistance analysis reveals GLUT‐1‐mediated glucose uptake as a major target of synthetic rocaglates in cancer cells
Source: Cancer Med. 2021 Sep 20;10(19):6807–22. doi: 10.1002/cam4.4212 (PMC8495295; doi:10.1002/cam4.4212)
Supplement: Supplementary file 1 — Supplementary Material [file CAM4-10-6807-s001.docx]

# Supplementary Figures


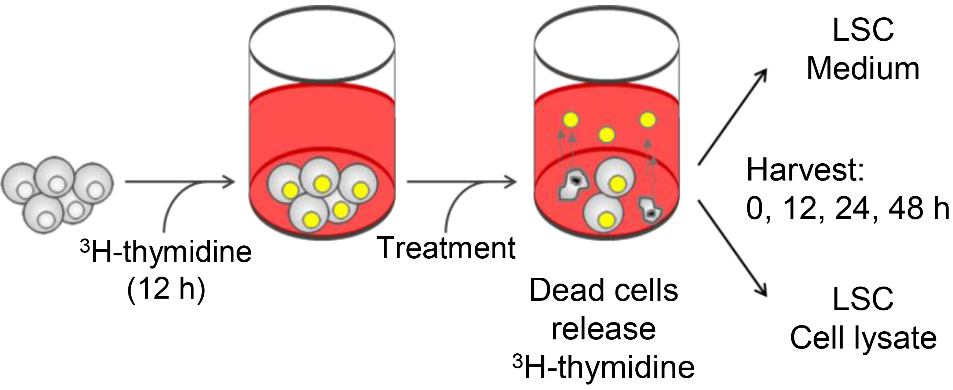


S1 Figure: Schematic depiction of pulse-chase cytotoxicity assay. Cells were labeled with ^3^H-thymidine for 12 h. Afterwards cells were treated with synthetic rocaglates for 0, 12, 24 and 48 h. Radioactive tracer was released from dying cells into the cell culture medium. At the end of each treatment period, medium was collected separately from the cellular lysate and both were subjected to LSC.


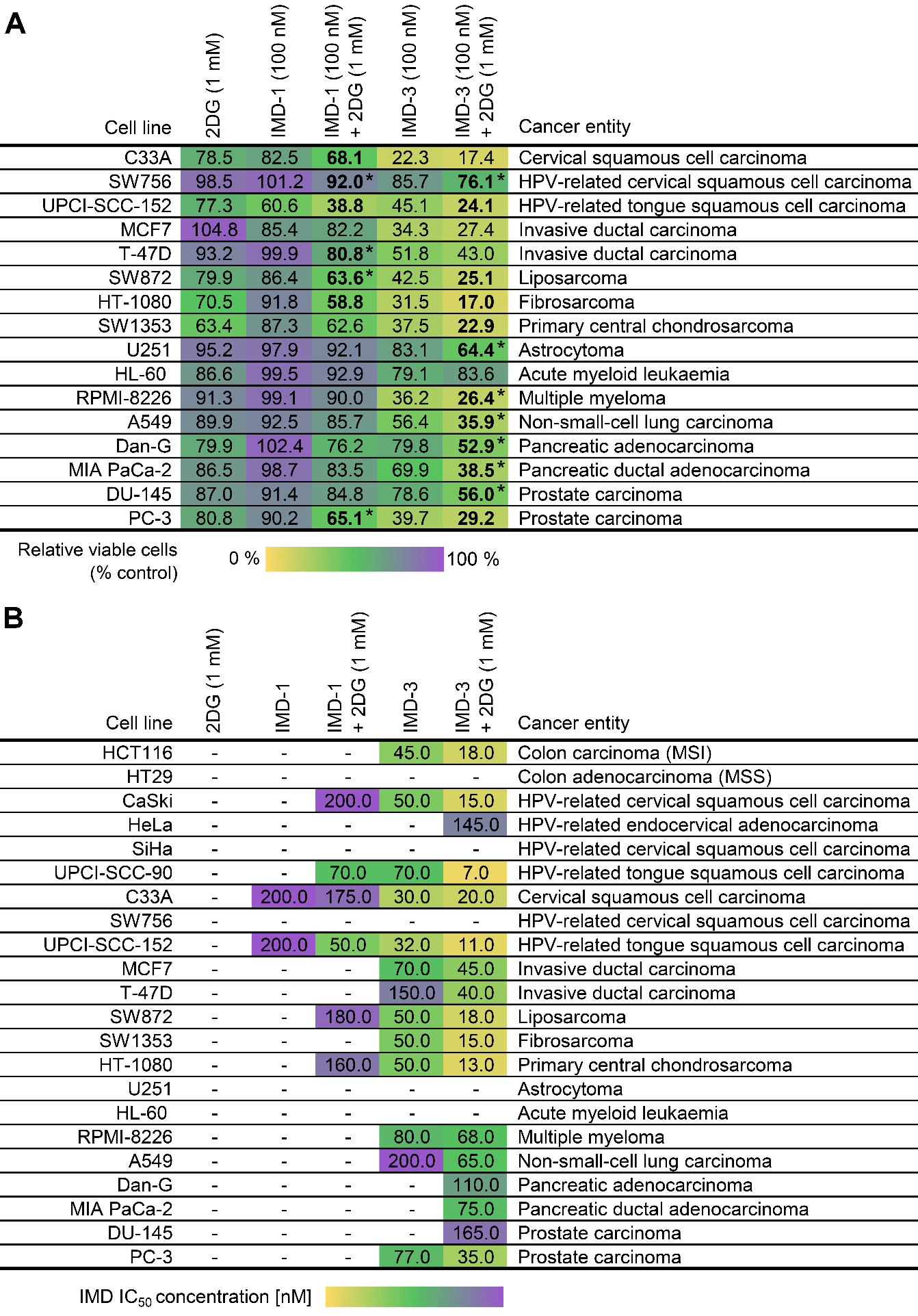


S2 Figure: Cell viability upon treatment with rocaglates (A) and IC_50_ concentrations of rocaglates alone and in combination with 2DG (1 mM) (B). (A) Cell viability upon 48 h treatment with synthetic rocaglates (IMD-1, IMD-3) alone and in combination with 2DG. Treatment combinations which were significantly more effective than both single treatments are highlighted in bold letters. Cell lines, in which enhanced growth-inhibitory effect upon combination of synthetic rocaglates with 2DG was observed, are marked with an asterisk. Significance was determined using two-way ANOVA and Bonferroni post-test. Cell lines derived from different tumor tissues including cervical (C33A, SW756), head and neck (UPCI-SCC-152), breast (MCF7, T-47D), soft tissue (SW872), mesenchyme (HT-1080), bone (SW1353), leukemia (HL-60, RPMI-8226), lung (A549), pancreatic (Dan-G, MIA PaCa-2), and prostate (Du-145, PC-3). Mean values of relative cell viability are shown. (B) IC_50_ values were determined using linear regression after application of different treatment concentrations (25, 50, 100, 200 nM) for 48 h.


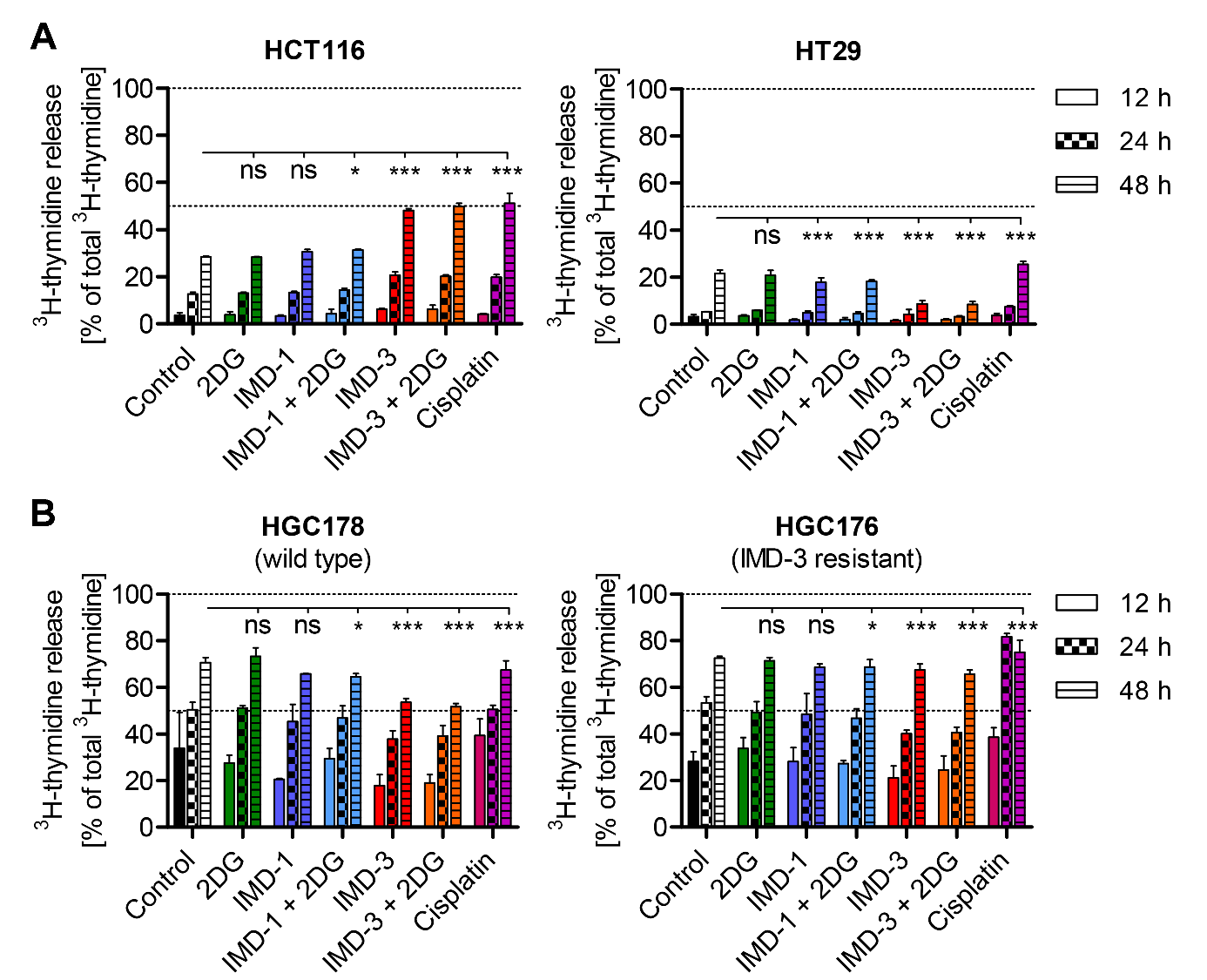


S3 Figure: Cytotoxicity of synthetic rocaglate treatment. (A) Release of ^3^H-thymidine (%) from labeled cells into the medium upon treatment for 12, 24 or 48 h in HCT116 and HT29 cells. Due to strong proliferation inhibition by IMD-3, release of ^3^H-thymidine is decreased in comparison to the control cells in HT29, HGC178 and HTC176 cells, therefore it was not possible to calculate cytotoxic cell death. ^3^H-thymidine release also revealed high turnover rate of HGC178 and HGC176 cells as the majority of the label was released into the medium after 48 h, although controls were approx. 95% confluent. (B) Release of ^3^H-thymidine (%) from labeled cells into the medium upon treatment for 12, 24 or 48 h in wild type HGC178 and IMD-3-resistant HGC176 cells. 2DG: 1mM, IMD-1: 100 nM, IMD-3: 100 nM, Cisplatin: 75 µM. * p ≤ 0.05; ** p ≤ 0.01; *** p ≤ 0.001; ns, non-significant. Only p-values for the 48 h time point are displayed in the graph.


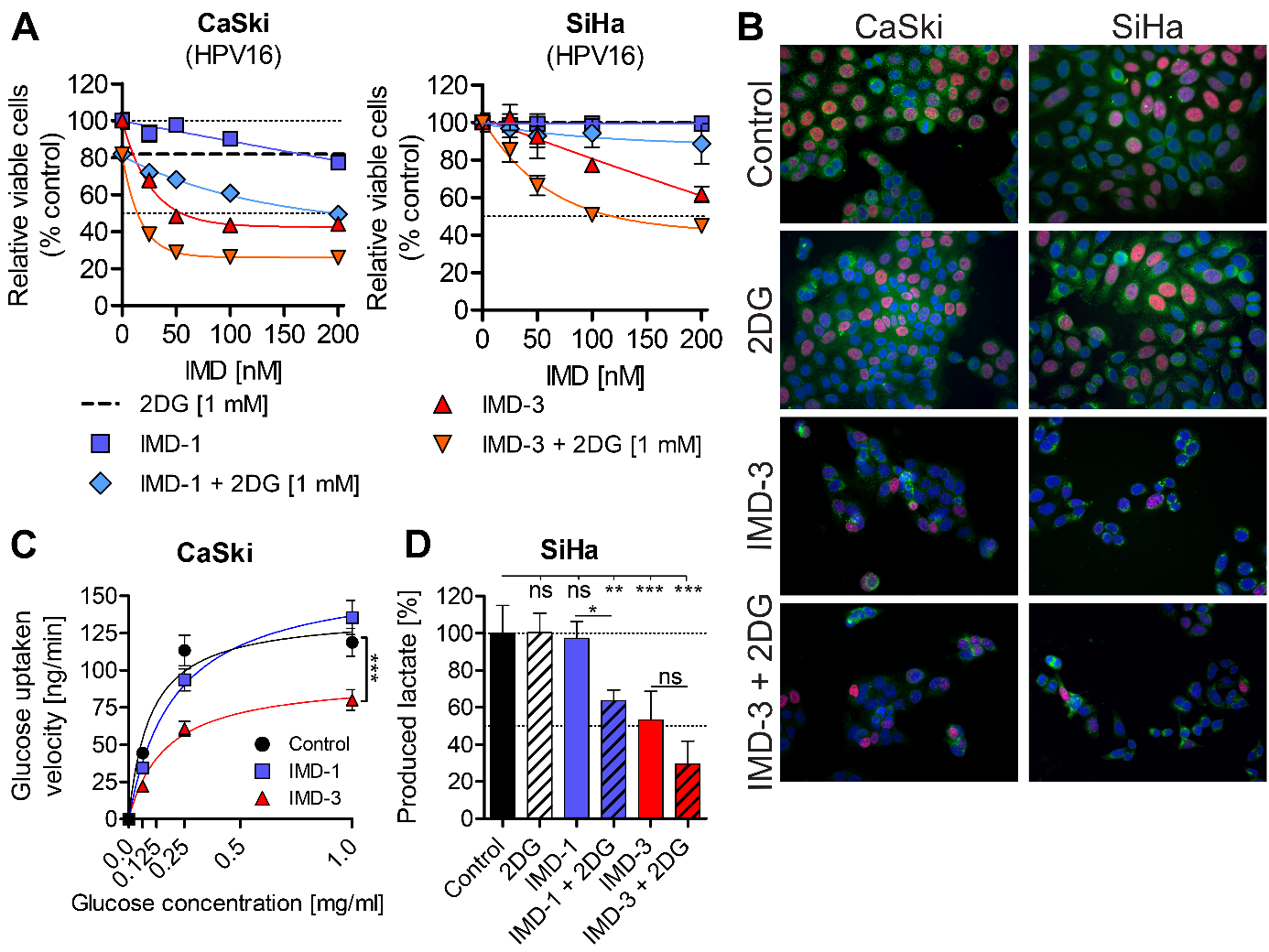


S4 Figure: Influence of synthetic rocaglates on glucose metabolism in HPV-transformed cervical cancer cell lines CaSki and SiHa. (A) Cell viability upon 48 h treatment with IMD-1 or IMD-3 alone and in combination with 2DG (1 mM) in CaSki and SiHa cells. (B) Immunofluorescent staining of glucose transporter GLUT-1 (green) and the proliferation marker EdU (red) shows reduced GLUT-1 expression and proliferation inhibition by IMD-3 treatment of cancer cells. Nuclei were DAPI stained. Magnification: 400-fold. (C) Glucose uptake velocity in CaSki cells upon synthetic rocaglate treatment (24 h) dependent on the glucose concentration in the medium using radioactive ^3^H-2DG. (D) Amount of secreted lactate by IMD treated SiHa cells after 24 h of treatment. * p ≤ 0.05; ** p ≤ 0.01;
*** p ≤ 0.001; ns, non-significant. 2DG: 1mM, IMD-1: 100 nM, IMD-3: 100 nM, Cisplatin: 75 µM.


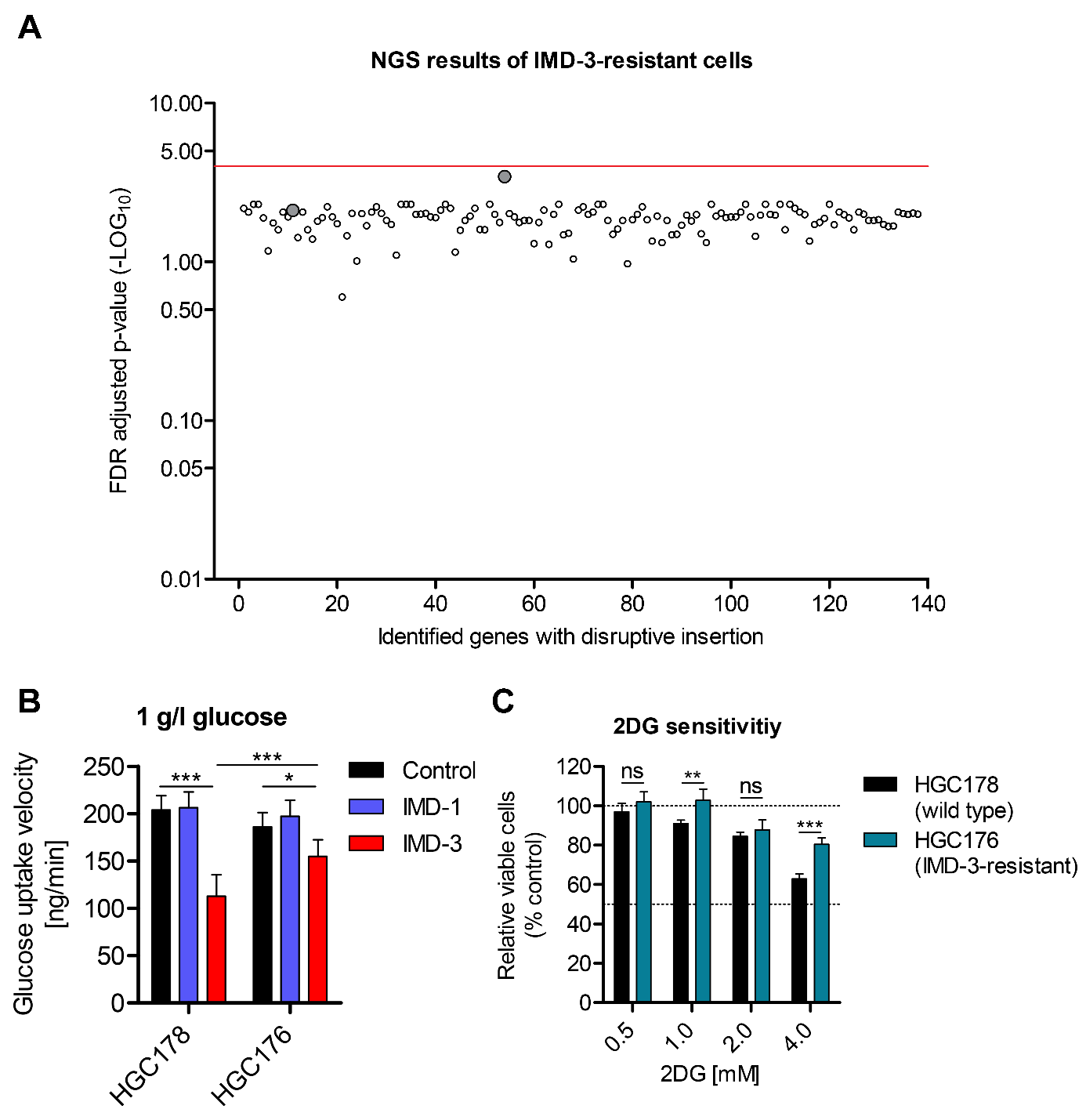


S5 Figure: Next generation sequencing (NGS) screening of gene-trapped IMD-3-resistant cell clones and influence of IMD-3 resistance on glucose uptake and sensitivity towards 2DG treatment. (A) NGS results of IMD-3-resistant gene-trap clones. One disruptive insertion was identified for 136 genes, while two genes exhibited two disruptive insertions (grey). No gene was significantly overrepresented (above red line: FDR adjusted p-value = 0.0001) indicating its importance for resistance development. Screening was performed by Haplogen GmbH (Vienna, Austria). (B) Glucose uptake velocity at 1 g/l glucose medium concentration. IMD-3 reduced the uptake velocity in both cell lines, however the effect was significantly stronger in sensitive HGC178 cells compared to resistant HGC176 cells. (C) Decreased sensitivity of IMD-3-resistant cells (HGC176) towards 2DG due to downregulation of glycolysis. * p ≤ 0.05; ** p ≤ 0.01; *** p ≤ 0.001; ns, non-significant. 2DG: 1mM, IMD-1: 100 nM, IMD-3: 100 nM, if not indicated otherwise; Cisplatin: 75 µM.


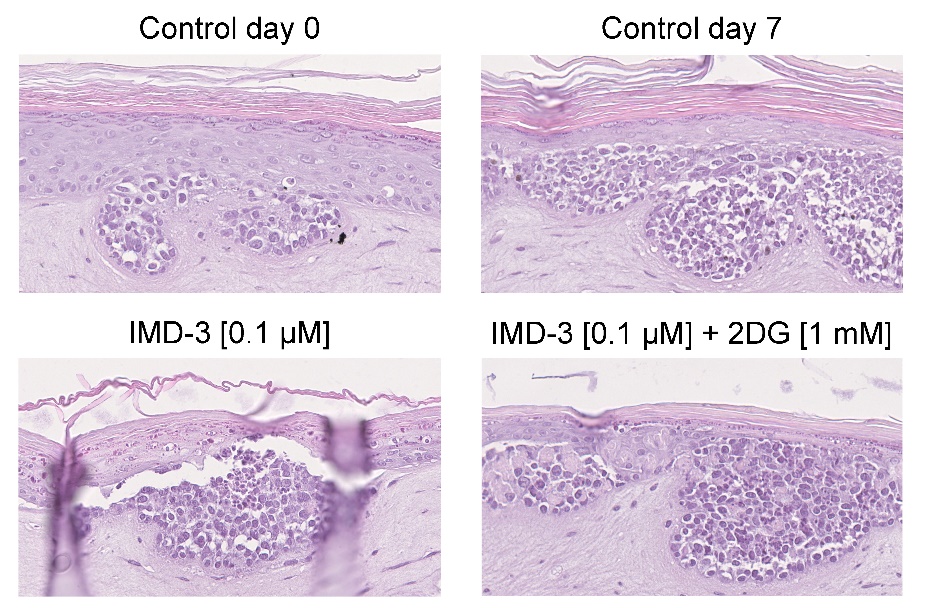


S6 Figure: Growth of tumor cell clusters in OTCs of SiHa tumor cells and normal keratinocytes under treatment with 2DG and a lower concentration of synthetic rocaglates (0.1 µM). Control cultures were fixed before treatment start (day 0) and 7 days after the first treatment and are also shown in Figure 6. The treatment with 0.1 µM IMD-3 alone or in combination with 2DG did not influence viability of tumor cells.

# Supplementary Methods

**Cell line screening**

Cell lines from different cancer entities were obtained from ECACC and cultured in: DMEM + 10% FBS + 1% P/S (A549 (RRID:CVCL_0023), C33A (RRID:CVCL_1094), Du-145 (RRID:CVCL_0105), HT-1080 (RRID:CVCL_0317), MCF7 (RRID:CVCL_0031), MIA PaCa-2 (RRID:CVCL_0428), PC-3 (RRID:CVCL_0035), SW756 (RRID:CVCL_1727), SW1353 (RRID:CVCL_0543), SW872 (RRID:CVCL_1730), T-47D (RRID:CVCL_0553), U251 (RRID:CVCL_0021)), RPMI1640 + 10% FBS + 1% P/S (Dan-G (RRID:CVCL_0243), HL-60 (RRID:CVCL_0002), RPMI-8226 (RRID:CVCL_0014)), RPMI1640 + 10% FBS + 0.05% gentamycin + 1% MEM-NEAA (UPCI-SCC-152 (RRID:CVCL_C058)).

**Lactate production – Medium processing**

Medium processing to measure secreted lactate concentration was performed as described in the following. 500 µl medium from treated cells were mixed with 400 µl 0.6 M perchloric acid and incubated on ice for 10 min to precipitate interfering proteins. Samples were centrifuged (10 min, 2°C, 16,000 g) and supernatant was mixed with 121 µl 3 M KOH and centrifuged again (10 min, RT, 16,000 g). 200 µl of the obtained supernatant was mixed with 334 µl glutamate buffer (0.8 M, pH 8.9), 50 µl β-NAD solution (18.75 mg/ml), 25 µl GPT (from pig heart, 100 U/ml in 1.8 M (NH_4_)_2_SO_4_) and 980 µl water in a cuvette and incubated (10 min, RT). Absorption was measured at 339 nm (A1) before adding 100 µl L-LDH (from bovine heart, 500 U/ml in 3.2 M (NH_4_)_2_SO_4_). After incubation (30 min, RT), absorption (A2) was measured and lactate concentration of the sample was calculated using the Lambert-Beer law.

**Transcriptome analysis**

Raw experiment data was imported from IDAT files provided by the DKFZ Genomics and Proteomics Core Facility along with sample information using the “beadarray” package[^24^](#_ENREF_24). Expression data was quantile normalized and log2-transformed using the “neqc”-method in the “limma” package[^25^](#_ENREF_25) and the resulting dataset was combined with probe annotation data contained in the “illuminaHumanv4.db” annotation package. Known bad quality and non-matching probes as described in the annotation were dropped from the result set. Differential expression between conditions was analyzed by fitting linear models and calculating empirical Bayes statistics with “limma” functions lmFit() and eBayes()[^24^](#_ENREF_24). Log2-fold changes (FCs) and Benjamini-Hochberg (BH)-adjusted p-values were obtained for all retained probes and annotated with Illumina’s probe identifier, common gene symbol and name and EntrezID and exported to CSV format. The obtained lists of differentially expressed genes (DEGs) were filtered based on their BH-adjusted p-value (p-value-BH ≤ 0.05) and a minimum FC of 2 (up- or downregulated). FCs of genes with several Illumina probes were averaged and the highest (most conservative) BH-adjusted p-value was used.

**Gene-trap mutagenesis of haploid cells**

To generate GT mutagenized IMD-3-resistant cells,293T cells were cultured in DMEM (+ 20% FBS, 1% P/S) and used for virus generation. Retroviral transfection was performed in fresh medium using Turbofectin including a GeneTrap vector containing GFP. After 48 h and 72 h, viral supernatant was collected and concentrated for infection of haploid Hap1 cells. Hap1 cells were incubated overnight with the virus. After 48-72 h Hap1 cells were checked for GFP expression by FACS (>70% positive). Gene-trap resistance screen for IMD-3 was performed by exposing gene-trapped and wild type (WT) Hap1 cells to 20 nM IMD-3 over the duration of one month. Afterwards the cells were exposed to 40 nM IMD-3 for one week before WT and GT cells were harvested. IMD-3-resistant GT cells were used to establish the resistant cell line HGC176 from the original WT cell line Hap1 (HGC178). Finally, DNA was extracted from WT and GT cells using QIAmp DNA Mini kit and 4 µg DNA was digested using MseI (37°C, overnight). DNA was purified (QIAquick DNA purification kit) and religation was performed using T4 DNA ligase. Again, DNA was purified and subjected to inverse PCR (iPCR) using following primers: CAAGCAGAAGACGGCATACGAGATACGAATTCGTGACTGGAGTTCAGACGTGTGCTCTT CCGATCgttctgtgttgtctctgtctG (forward); ATGATACGGCGACCACCGAGATCTACACTAATCTTAACACTCTTT CCCTACACGACGCTCTTCCGATCTatctgatggttctctagcttgcc (reverse). Resulting DNA was analyzed by next generation sequencing (NGS) as described previously[^26^](#_ENREF_26).

**HE staining**

HE staining was performed following standard protocols. Briefly, slides were deparaffinized, rehydrated, washed with deionized water and stained with hematoxylin. After washing the slides in tab water, eosin staining was performed, slides were washed with deionized water and after an incubation in a solvent series (water + glacial acetic acid, 70% EtOH, 96% EtOH, 100% EtOH, xylene (2x)) mounted with histofluid. Stained slides were scanned using a NanoZoomer S210 Digital slides scanner with a 40x objective (Hamamatsu Photonics K.K., Hamamatsu, Japan).
